# Effects of Combined Aerosolization with Ultraviolet C Light-Emitting Diode on Enterohemorrhagic *Escherichia coli* and *Staphylococcus aureus* Attached to Soft Fresh Produce

**DOI:** 10.3390/foods10081834

**Published:** 2021-08-08

**Authors:** Chae-Lim Lee, Geun-Hyang Kim, Ki-Sun Yoon

**Affiliations:** Department of Food and Nutrition, College of Human Ecology, Kyung Hee University, Dongdaemun-gu, Seoul 02447, Korea; crl95@khu.ac.kr (C.-L.L.); dytnfvos@naver.com (G.-H.K.)

**Keywords:** slightly acidic electrolyzed water (SAEW), pathogen populations, reduction effect, moisture loss, visual quality

## Abstract

Washing soft fresh produce such as strawberries, baby leaves, and sliced onions with sanitizing agents is challenging due to their fragile texture. Thus, treatments like aerosolization using slightly acidic electrolyzed water (SAEW) and ultraviolet C light-emitting diode (UVC LED) irradiation may be good alternatives. In the present study, the reduction effects of a combined treatment of aerosolization using SAEW and UVC LED irradiation on enterohemorrhagic *Escherichia coli* (EHEC) and *Staphylococcus aureus* attached to strawberries, baby leaves, and sliced onions were investigated. The behaviours of EHEC and *S. aureus*, moisture loss, colour measurement, and visual appearance were also analyzed at 10 and 15 °C for 7 days. The reduction effect of the combined treatment with 100 SAEW and UVC LED was higher (0.53–0.92 log CFU g^−1^) than a single aerosolization treatment (0.11–0.41 log CFU g^−1^), regardless of samples or pathogens. A greater effect on EHEC and *S. aureus* reduction was observed in strawberries (0.74 and 0.92 log CFU g^−1^) than in baby leaves (0.62 and 0.53 log CFU g^−1^) and sliced onions (0.55 and 0.62 log CFU g^−1^). The combined treatment further reduced the EHEC and *S. aureus* populations in strawberries during 7 days of storage at 10 and 15 °C. However, the EHEC and *S. aureus* populations were maintained in baby leaves and sliced onions at 10 °C for 7 days. Additionally, the greatest effect on the maintenance of colour and appearance was obtained in the combined treatment. Since the combined treatment reduces EHEC and *S. aureus* populations and preserves visual quality, it could be expected to extend the shelf life of soft fresh produce at the retailer stage of the supply chain.

## 1. Introduction

The fresh-cut produce market has increased drastically to meet consumer demand for healthy and ready-to-eat foods. The production of fresh-cut produce showed an increase of 64.8% from 2013 to 2017 in Korea [1]. Likewise, the market for meal kits is also on the rise, since the meal kit industry was valued at 4.65 billion US dollars in 2017, representing a 300% growth in the United States [2]. Both fresh-cut produce and some meal kit products consist of various vegetables that can be consumed as non-processed produce.

A total of 515 cases and six deaths due to foodborne outbreaks were reported in the United States from 2011 to 2020 due to enterohemorrhagic *Escherichia coli* (EHEC) in fresh produce [3,4,5]. Specifically, 165 cases and 2 deaths due to EHEC in a salad were reported in 2016 in the UK and 235 cases due to EHEC in onion were reported in 2008 in Canada [6,7]. In Korea, *Staphylococcus aureus* has been regularly detected in fresh and fresh-cut produce at the market. *S. aureus* was detected in 1 out of 181 baby-leaf vegetables (0.6%) [8], four out of 60 tomatoes (6.7%), one out of 60 mandarins (1.7%) [9], six out of 390 fresh ready-to-eat food (1.5%) [10], one out of 150 lettuces (0.7%), two out of 150 cabbages (1.3%), and one out of 150 celeries (0.7%) [11].

The sanitizing step in preparing fresh produce is essential to reduce the risk of foodborne illness. Washing soft fruits, baby leaves, and sliced onions might lead to the loss of their commercial value due to changes in shape and appearance because they have fragile structures. They may have a higher microbial growth incidence after the postharvest period without a washing step [12,13,14,15,16]. Aerosolization of sanitizers could reduce the population of foodborne pathogens on soft fresh produce at the market [17,18,19,20]. Aerosols are defined as clumps of solid or liquid particles dispersed in the air, typically from 0.001 to 100 μm in size. Thus, aerosolization converts sanitizer into fine mist particles, and it could be applied to disinfect produce with fragile structures in its surfaces.

Ultraviolet (UV) light is a non-thermal method of disinfection that induces the formation of pyrimidine dimers in the DNA and RNA of a microorganism, thus interfering with DNA replication and ultimately leading to cell death [21,22]. UV irradiation is used with low-pressure mercury (LPM) lamps which contain glass and mercury, which represent both physical and chemical hazards in food production environments [23]. The Minamata Convention on Mercury treaty also prohibits the manufacture, import, and/or export of mercury-containing products [24]. In this sense, ultraviolet C light-emitting diodes (UVC LED) could compensate for the several limitations of LPM lamps. Some advantages of UVC LED include their small size, robustness, lack of mercury, long lifetime, no pre-heating, and the ability to operate effectively at cold temperatures [25,26,27]. It has been shown that UVC LED can effectively reduce pathogen populations in fresh-cut produce [28].

Several studies have also investigated the effects of aerosolization, UV LED, or UVC LED on reducing bacteria populations in food [17,19,29,30,31,32]. Jiang et al. [17] suggested an in-package aerosolization technology to improve the microbial safety of cherry tomatoes against *Salmonella* spp. Aerosolized malic acid was also suggested as an alternative sanitizer to increase the microbial safety of fresh produce such as spinach and lettuce [19]. The combined effect of slightly acidic electrolyzed water (SAEW) and UV LED has better decontamination efficiency against *Salmonella* spp. and *E. coli* in coriander than a single treatment [29]. An inactivation effect of UVC LED treatment on *E.* *coli* O157:H7, *S.* Typhimurium, and *L.* *monocytogenes* inoculated onto sliced deli meat, spinach surface [30] and sliced cheese [32] were reported. However, the combined effect of the aerosolization of malic acid and UVC on the inactivation of pathogens in fresh-cut lettuce was only reported by Seong et al. [31]. As far as we know, the combined effect of aerosolization using SAEW and UVC LED for soft fresh produce has not been reported yet. Therefore, the main objectives of this study were (1) to investigate the efficacy of the combined treatment of aerosolization using SAEW and UVC LED (275 nm) in order to reduce bacteria on soft fresh produce, including strawberries, baby leaves, and sliced onions, and (2) to determine the applicability of combined treatment on an industrial scale through the evaluation of quality.

## 2. Materials and Methods

### 2.1. Bacteria Strains

Enterohemorrhagic *Escherichia coli* (EHEC) strains, including *E. coli* O157:H7 (NCTC 12079) and non-O157 enterohemorrhagic *E. coli* (NCCP 13720, 13721), were obtained from the Ministry of Food and Drug Safety (MFDS) in Korea. Enterotoxin A-producing *S. aureus* (SEA; ATCC 13565) was purchased from American Type Culture Collection (ATCC). Enterotoxin G and I-producing *S. aureus* and non-enterotoxin-producing *S. aureus* were isolated from red cabbage and pineapple, respectively, which were obtained at the market. Each stock culture of EHEC and *S. aureus* was maintained at −80 °C in tryptic soy broth (TSB, MBcell, Seoul, Korea) containing 20% glycerol.

### 2.2. Culture Preparation

Each strain of EHEC and *S. aureus* was cultured in 10 mL of sterile TSB and incubated at 36 °C for 24 h in a rotary shaker (VS-8480SP, Vision, Daejeon, Korea) at 140 rpm. To prepare cocktail cultures of each bacterium, an equal quantity of each strain was combined and added into a sterilized 50 mL conical tube (SPL Life Science, Pocheon, Korea). For the bacterial inoculum solution, the cocktail suspension was serially diluted 10-fold with 0.1% peptone water (PW, Difco, Detroit, MI, USA). Then, 10 mL of the diluted cocktail suspension was mixed with 990 mL of sterile 0.1% PW in a sterile beaker of 2 L.

### 2.3. Sample Preparation and Inoculation

A pack of baby leaves (150 g), strawberries (500 g), and a bag of onion (5 onions) were purchased from the supermarket (Seoul, Korea) for each experiment. Each pack of baby leaves consisted of a mix of tatsoi, bok choy, and radish sprouts. All samples were visually selected and stored at 4 °C until use. To remove indigenous microorganisms, baby leaves were submerged in 3.6% hydrogen peroxide for 5 min, according to the protocol described by Kim et al. [33], rinsed with tap water for 2 min, and then exposed to UVC LPM lamp irradiation for 30 min. After removing indigenous microorganisms, 100 g of baby leaves were dipped into a bacterial inoculum solution by stirring with a sterile magnetic stir bar for 2 min. These inoculated samples were air-dried on a sterile stainless-steel tray under an aseptic bench for 30 min. To prepare sliced onions, onions were peeled and aseptically cut into slices with a stainless-steel knife and then dipped in a bacterial inoculum solution in the same way as the baby leaf vegetables. Approximately 10 g of each strawberry was hulled and washed with tap water. A total of 100 µL of prepared cocktail suspensions was spot inoculated onto strawberries and air-dried in a sterile stainless-steel tray under an aseptic bench for 30 min. After the air-drying process, 100 g of each sample was transferred to a new sterile stainless tray for aerosolization treatment with sanitizers.

The initial inoculum levels of strawberries, sliced onions, and baby leaves were 5–6 log CFU g^−1^.

### 2.4. Microbial Reduction Procedure of Soft Fresh Produce

#### 2.4.1. Procedure of Aerosolization Treatment with Sanitizers

Sodium hypochlorite (100 NaClO, Hanson Hygiene Co., Cheonan-si, Korea) solution at 100 ppm was prepared according to the manufacturer’s instructions. Both 60 ppm of slightly acidic electrolyzed water (60 SAEW) and 100 ppm of SAEW (100 SAEW) were provided by Cosmic Round Korea Co., Seongnam, Korea. The pH and available chlorine concentrations of sanitizers were measured with a pH meter (Orion-star pH-benchtop, Thermo, Waltham, MA, USA) and chlorine test papers (Toyo Roshi Kaisha, Ltd., Tokyo, Japan), respectively.

The aerosolization test chamber was made of 5 mm-thick clear acryl sheets (460 × 460 × 300 mm). The chamber was sealed, and aerosolized mist was generated using an ultrasonic nebulizer (402 AI, Yuwell Medical Equipment & Supply Corp., Suzhou, China). The aerosolized mist was transferred from the machine through a tube to the lid of the chamber. The nebulizer could atomize sanitizers into droplets with a diameter of approximately 3.9 to 5 µm. Inoculated samples were treated with aerosolized sanitizers for 10 min in the chamber. After the treatment, the samples were transferred to an aseptic bench and air-dried for 30 min.

#### 2.4.2. Procedure of Ultraviolet C Light-Emitting Diode (UVC LED) Irradiation Treatment

A total of ten ultraviolet C light-emitting diode (UVC LED) modules (Bluelumi, Yongin-si, Korea) were set in a chamber (500 × 400 × 520 mm) equipped with two PCBs. Five UVC LED chips (275 nm wavelength) were linearly connected on an electronic printed circuit board (PCB; 450 × 25 mm). UVC LED irradiation was performed at a distance of 6 cm between the UVC LED and the samples used in the present study. The irradiance intensity of the UVC LED module was measured with a spectrometer (UVC-254SD, Lutron Electronics Co., Inc., Coopersburg, PA, USA) previously calibrated at a range of 240–390 nm. The UVC radiation intensity was 173 µWcm^−2^.

Aerosolized and air-dried soft fresh produce (strawberries, baby leaves, and sliced onions, 10 g each) were transferred to polyethylene terephthalate (PET) containers (DA-0604, Modenpojang, Seoul, Korea). Thus, each sample was placed in the UVC LED chamber and was irradiated for 3 min with UVC LED doses of 31.14 mJ cm^−2^.

### 2.5. Microbial Analysis

Treated soft fresh produce (10 g) was transferred into a sterile stomacher bag (JS-011, Jinsung Uni-Tech Co., Ltd., Goyang-si, Korea) and homogenized with 90 mL of sterile 0.1% peptone water (PW, Becton, Dickinson and Company, Sparks, NV, USA) using a stomacher (BagMixer, Interscience, St Nom la Bretêche, France) for 2 min. The homogenized solution was serially diluted 10-fold with sterile 0.1% PW and spread onto non-selective and selective agar plates for each pathogen. Eosin methylene blue agar (EMB, Oxoid, Hampshire, UK) and Baird–Parker agar bases (BPA, MBcell, Seoul, Korea) were used as selective media to enumerate EHEC and *S. aureus* populations, respectively. Tryptic soy agar (TSA, MBcell, Seoul, Korea) was used to enumerate both EHEC and *S. aureus* populations as a non-selective medium. Plates were incubated for 24–48 h at 37 °C. Every bacterial growth enumeration was based on duplicate counts.

### 2.6. Quality Evaluation during Storage at 10 and 15 °C after the Combined Treatment

#### 2.6.1. Behaviors of EHEC and *S. aureus* on Soft Fresh Produce during Storage

Behaviors of EHEC and *S. aureus* were compared among samples, including samples not washed with sanitizer (controls), samples treated with aerosolization at 100 ppm SAEW (100 SAEW), and samples treated with aerosolization at 100 ppm and irradiated with UVC LED for 3 min (100 SAEW + UVC LED). Each sample (10 g) was collected into a PET container with a lid and stored for 7 days at 10 (storage temperature) and 15 °C (abused temperature). EHEC and *S. aureus* populations were enumerated at intervals of 24 h during the 7-day period using the selective media.

#### 2.6.2. Moisture Loss Analysis

Moisture losses of control and treated (100 SAEW, 100 SAEW + UVC LED) samples were measured every 24 h during the 7 days of storage at 10 and 15 °C. This parameter was calculated as the difference between the initial weight and the weight at the sampling date using the following formula:

Moisture loss (%) = (initial weight − sampling date weight) / initial weight × 100(1)

#### 2.6.3. Colour Measurement and Visual Appearance Evolution

The colour of each sample was measured every 24 h during the 7 days of storage at 10 and 15 °C using a colorimeter (Minolta CR-400, Osaka, Japan). The colour was described as coordinates: L*, a*, and b* colour values. The L* parameter shows lightness to darkness evolution. It ranges from 0 (black) to 100 (white). The a* parameter measures the degree of redness (+a*) or greenness (−a*). The b* parameter measures the degree of yellowness (+b*) or blueness (−b*). The evolution of the visual appearance of controls and treated (100 SAEW, 100 SAEW+UVC LED) samples were photographed daily for 7 days at 10 and 15 °C. The most distinct changes of each sample’s properties that were observed include browning and molding in strawberries, yellowing and softening of baby leaves, and browning and dryness of sliced onions.

### 2.7. Statistical Analysis

All experiments were repeated twice, with three replicates per treatment. Statistical analyses were conducted with Statistical Analysis System (SAS) version 9.4 (SAS Institute Inc., Cary, NC, USA). Statistical comparisons for more than two groups were performed using one-way analysis of variance (ANOVA) followed by Duncan’s test for multiple range tests. A value of *p* < 0.05 was considered statistically significant.

## 3. Results and Discussion

### 3.1. Effect of Aerosolization Alone and Combined Treatment with UVC LED on the Reduction in EHEC and S. aureus

**Figure 1:**
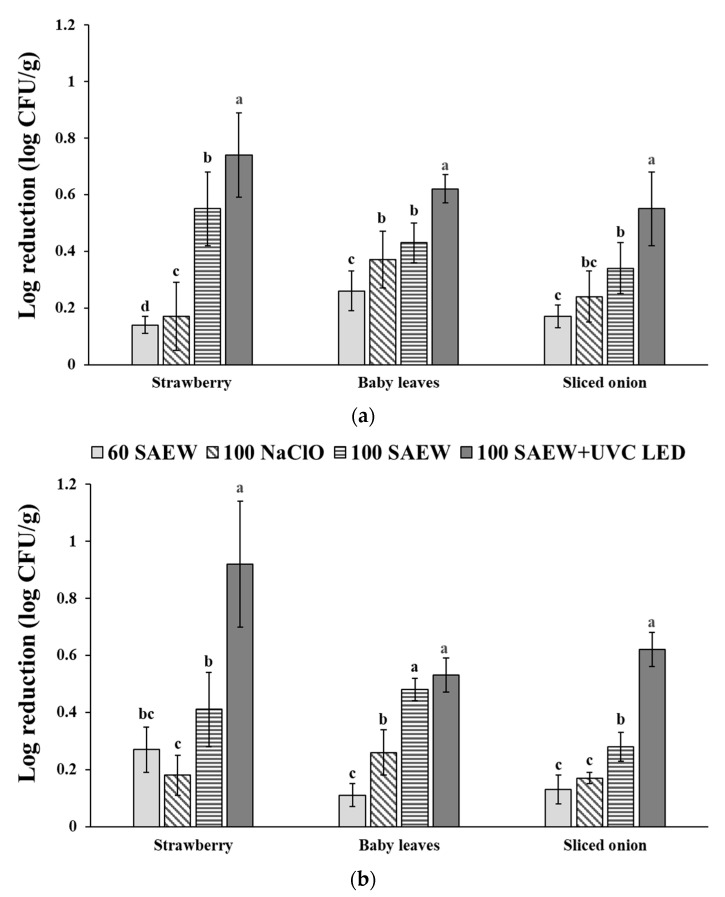
Comparison of log reduction in aerosolization alone and combined treatments with UVC LED on (**a**) enterohemorrhagic *E. coli* (EHEC) and (**b**) *S. aureus* in strawberry, baby leaves, and sliced onion. 60 SAEW, aerosolization with 60 ppm SAEW; 100 NaClO, aerosolization with 100 ppm NaClO; 100 SAEW, aerosolization with 100 ppm SAEW; 100 SAEW + UVC LED, aerosolization with 100 ppm SAEW and radiated UVC LED (275 nm) for 3 min. ^a–d^ Different letters represent significant differences measured by Duncan’s multiple tests among treatments within each soft fresh produce at *p* < 0.05.

The effects of aerosolization with 60 SAEW, 100 SAEW, or 100 NaClO and combined treatment of 100 SAEW and UVC LED on the reduction in EHEC and *S. aureus* attached to strawberries, baby leaves, and sliced onions were compared (Figure 1). Among single treatments used for aerosolization, the 100 SAEW was the most effective in reducing the microbial population of two pathogens in all three soft fresh produce. The highest reduction (0.34–0.55 log CFU g^−1^) of EHEC populations was observed after treatment with 100 SAEW, followed by that with 100 NaClO (0.24–0.33 log CFU g^−1^) and 60 SAEW treatment (0.14–0.26 log CFU g^−1^) (Figure 1a). The highest reduction (0.28–0.41 log CFU g^−1^) of *S. aureus* populations was also observed after treatment with 100 SAEW, followed similarly by that with 100 NaClO (0.17–0.26 log CFU g^−1^) and 60 SAEW treatment (0.11–0.27 log CFU g^−1^) (Figure 1b).

Cho et al. [34] reported that the aerosolization of carrots with 100, 200, 300, and 400 ppm chlorine dioxide (ClO_2_) for 10 min could reduce *E. coli* O157:H7 populations by 0.2–1.1 log CFU g^−1^, *Salmonella* Typhimurium by 0.2–1.4 log CFU g^−1^, and *L. monocytogenes* by 0.2–1.2 log CFU g^−1^. Aerosolization of spinach with 400 ppm peracetic acid solution for 15 min could achieve 0.97 ± 0.30 log CFU g^−1^, 1.02 ± 0.23 log CFU g^−1^, and 0.92 ± 0.20 log CFU g^−1^ reductions for *E. coli* O157:H7, *Salmonella* Typhimurium, and *L. monocytogenes*, respectively [35]. After aerosolization with 2% malic acid for 10 min, populations of *L. monocytogenes*, *Salmonella* Typhimurium, and *E. coli* O157:H7 populations were reduced by 0.72, 0.66, 1.03 log CFU g^−1^ in spinach and 0.83, 1.02, 1.35 log CFU g^−1^ in lettuce, respectively [19].

Generally, the reduction effect of aerosolization was lower than that of the dipping treatment commonly used to reduce the microbial population. Even when a lower concentration (ppm) and less washing time were applied, the dipping treatment showed a greater reduction effect than aerosolization. The *S. aureus* population was reduced by 1.42–1.65 log CFU g^−1^ when iceberg lettuce, chicory, and cabbage were immersed in 30 ppm SAEW for 5 min [36]. When carrot, celery, cabbage, and paprika were immersed in 100 ppm NaClO for 3 min, *E. coli* and *S. aureus* populations were reduced by 0.90–1.34 log CFU g^−1^ and 0.58–1.70 log CFU g^−1^, respectively [28]. Since aerosolization technology could be applied to produce with a soft texture that is difficult to clean [17], the concentration of sanitizer is generally increased to improve the reduction effect of aerosolization [17,34,35]. However, a high concentration of sanitizer could lead to consumers’ concerns due to its impact on the environment. Thus, incorporating another effective tool with the aerosolization of a sanitizer with a low concentration (<100 ppm) is needed for fragile produce, such as baby leaves and berries.

In this study, the combined treatment of 100 SAEW and UVC LED was more effective in reducing the populations of EHEC and *S. aureus* in strawberries than in baby leaves or sliced onions. The combined treatment of 100 SAEW and UVC LED reduced EHEC in strawberries, baby leaves, and sliced onions by 0.74 ± 0.15, 0.62 ± 0.05, and 0.55 ± 0.13 log CFU g^−1^, respectively (Figure 1a). These combined treatments reduced *S. aureus* populations in strawberries, baby leaves, and sliced onions by 0.92 ± 0.22, 0.53 ± 0.06, and 0.62 ± 0.06 log CFU g^−1^, respectively (Figure 1b). A greater reduction was observed for *S. aureus* than for EHEC in strawberries and sliced onions, whereas the reduction was significantly higher for EHEC than *S. aureus* in baby leaves (Figure 1). The results show that the combined treatment of 100 SAEW and UVC LED had a more efficient bactericidal effect than a single aerosolization treatment. In Korea, 84% of food manufacturing and processing industries use NaClO as a sanitizer, and dipping in 100 ppm NaClO solution for 5 min is the most commonly used method to sanitize fresh produce [37]. In this work, no significant difference in EHEC or *S. aureus* reduction was observed between dipping in 100 ppm NaClO for 5 min and the combined treatment of 100 SAEW and UVC LED treatment (data not shown). According to previous studies [29,31,38,39], combined treatment of washing methods with UVC LED or UVC LPM lamps could result in a more effective reduction in foodborne pathogens in fresh produce. Jiang et al. [29] revealed that a combined treatment of 60 ppm SAEW for 5 min with UV LED (240 µWcm^−*2*^ at 6 cm distance) for coriander reduced *Salmonella* spp. and *E. coli* populations by 2.42–2.72 log CFU g^−1^. This combined treatment was more effective than a single treatment with SAEW washing or UV LED, which reduced *Salmonella* spp. and *E. coli* populations by 1.8–1.85 log CFU g^−1^ and 1.1–1.2 log CFU g^−1^, respectively. Combined treatment of aerosolization with 2% malic acid for 20 min and UVC irradiation (254 nm) for 30 min was also more effective in reducing pathogen populations than a single treatment [31]. After combined treatment, populations of *E. coli* O157:H7, *Salmonella* Typhimurium, and *L. monocytogenes* in fresh-cut lettuce were reduced 1.65, 1.01, and 1.06 log CFU g^−1^, respectively. In contrast, their populations after a single treatment of aerosolization with 2% malic acid or UVC irradiation were reduced to 1.10, 0.53, and 0.70 or 1.22, 0.72, and 1.01 log CFU g^−1^, respectively. The population of *Salmonella* spp. in lettuce was reduced by 2.97 log CFU g^−1^ after combined treatment of 80 ppm SAEW dipping for 2 min and UVC LED with conditions of 100 µWcm^−2^ at 6 cm distance [38]. The effect of the combined treatment was greater than that of a single treatment with SAEW washing (1.42 log CFU g^−1^ reduction) or with UVC LED alone (1.58 log CFU g^−1^ reduction). In addition, combined treatment of 50 ppm ClO_2_ dipping for 5 min with UVC irradiation (254 nm) was more effective in reducing *E. coli* O157:H7 and *Salmonella* Typhimurium in romaine lettuce and kale than single treatments [39]. These results indicate that UVC LED is more effective when it is combined with other washing treatments. Thus, it could be used as another hurdle technology combined with other sanitizers in the fresh produce industry.

### 3.2. Effect of Combined Treatment of Aerosolization with UVC LED on Behaviours of EHEC and S. aureus in Soft Fresh Produce during Storage at 10 and 15 °C

**Figure 2:**
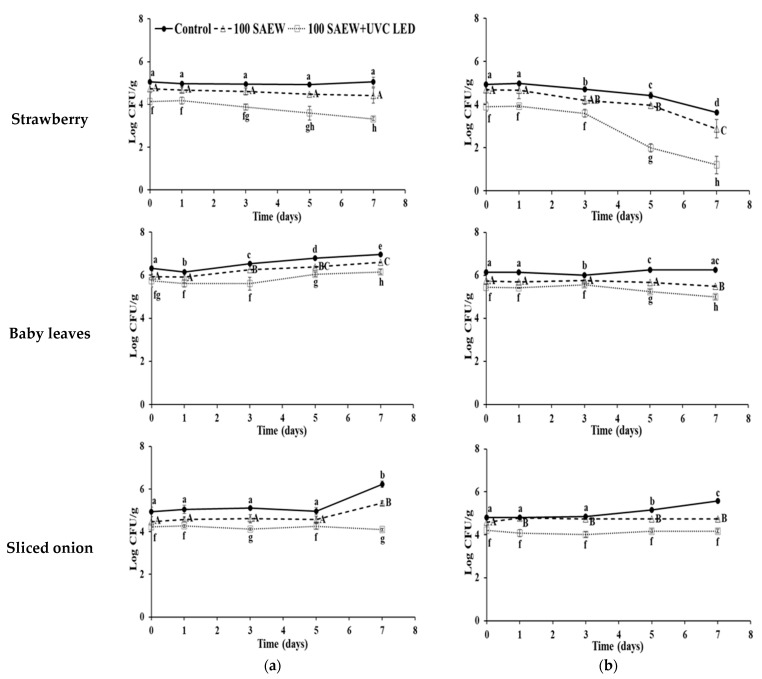
Log number of enterohemorrhagic *E. coli* (EHEC) (**a**) and *S. aureus* (**b**) after treatment in strawberry, baby leaves, and sliced onion stored for 7 days at 10 °C. ●, no extra wash with sanitizer (control); △, aerosolization with 100 ppm SAEW (100 SAEW); □, aerosolization with 100 ppm SAEW and UVC LED irradiation with 275 nm (100 SAEW + UVC LED). ^a–e; A–C; f–h^ Different letters within each treatment represent significant differences by Duncan’s multiple tests at *p* < 0.05.

**Figure 3:**
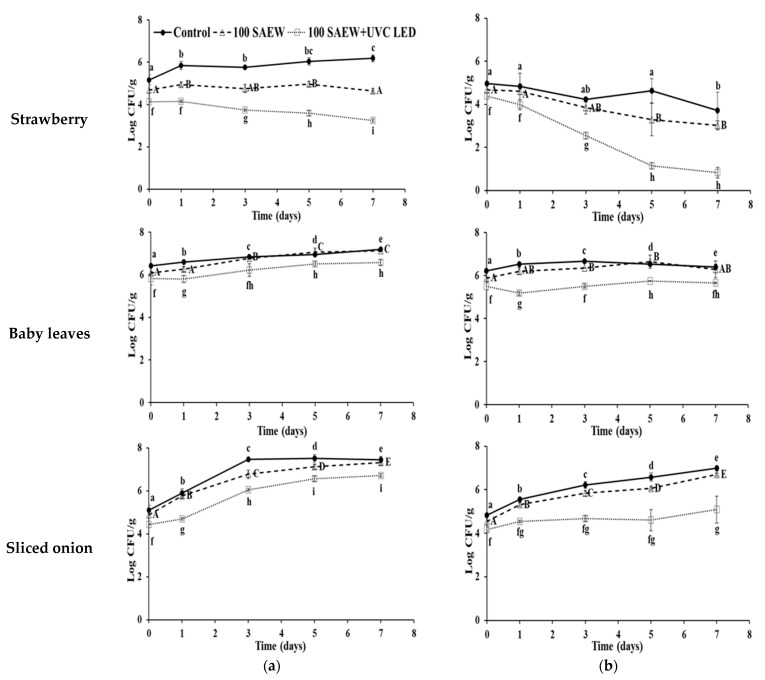
Log number of enterohemorrhagic *E. coli* (EHEC) (**a**) and *S. aureus* (**b**) after treatment in strawberry, baby leaves, and sliced onion stored for 7 days at 15 °C. ●, no extra wash with sanitizer (control); △, aerosolization with 100 ppm SAEW (100 SAEW); □, aerosolization with 100 ppm SAEW and UVC LED irradiation with 275 nm (100 SAEW + UVC LED). ^a–e; A–E; f–i^ Different letters within each treatment represent significant differences by Duncan’s multiple tests at *p* < 0.05.

The effects of control, 100 SAEW, and 100 SAEW with UVC LED on populations of EHEC and *S. aureus* were investigated after storage at 10 and 15 °C for 7 days (Figure 2 and Figure 3). No extra wash with a sanitizer was used for comparison as a control.

At 10 °C, the population of EHEC in the strawberry control group and 100 SAEW group was maintained, whereas the population of EHEC decreased by 0.8 log CFU g^−1^ in strawberries treated with 100 SAEW and UVC LED during the 7 days of storage (Figure 2a). On the other hand, the growth of EHEC in baby leaves was not inhibited in any of the treatment conditions. For sliced onions, the control group, 100 SAEW group, and combined group with 100 SAEW and UVC LED maintained the populations of EHEC for 5 days of storage. The control group and the 100 SAEW group showed increases of 0.87–1.3 log CFU g^−1^ on the EHEC population after 5 days of storage, whereas the combined treatment group inhibited the growth of EHEC and decreased its population at this point of storage.

At 10 °C, the population of *S. aureus* in strawberries showed decreases in all treatment conditions (Figure 2b). The population of *S. aureus* in strawberries showed a more rapid reduction with combined treatment of 100 SAEW and UVC LED than control or 100 SAEW treatment. The combined treatment reduced *S. aureus* population in strawberries the most (by 2.7 log CFU g^−1^) after 7 days of storage, followed by 100 SAEW (1.82 log CFU g^−1^) and control (1.3 log CFU g^−1^). The groups of 100 SAEW and combined treatment of 100 SAEW and UVC LED inhibited the growth of *S. aureus* in baby leaves and sliced onion during storage. However, increased growth of *S. aureus* (about 0.77 log CFU g^−1^) in sliced onion was observed in the control group.

At 15 °C, the growth of EHEC in strawberries was prevented with 100 SAEW and combined treatment of 100 SAEW and UVC LED, whereas a 1.0 log CFU g^−1^ increase in EHEC in strawberries was observed in the control group after 7 days of storage (Figure 3a). However, the growth of EHEC in baby leaves and sliced onions was not inhibited in any of the treatment conditions.

On the other hand, populations of *S. aureus* in strawberries were decreased after 7 days of storage in all treatment conditions (Figure 3b). The combined treatment of 100 SAEW and UVC LED resulted in the most significant reduction in *S. aureus* population in strawberries (3.57 log CFU g^−1^), followed by 100 SAEW (1.64 log CFU g^−1^) and control (1.26 log CFU g^−1^). Growth of *S. aureus* in baby leaves and sliced onions was observed in all treatment conditions. However, *S. aureus* in both baby leaves and sliced onions showed the least growth with 100 SAEW and UVC LED treatment. These results indicate that the combined treatment could be an effective tool to extend shelf life for soft fresh produce and meal kit vegetables such as sliced onions.

Several previous studies have reported different behaviors of foodborne pathogens in fresh produce during storage after a reduction treatment [17,28,29,31,40,41,42]. Growths of *E. coli* and *S. aureus* in carrots and celery have been observed after treatment with a combination of 30 ppm SAEW, ultrasound, and UVC LED when they are stored at 15 °C for 7 days [28]. Likewise, Jiang et al. [29] reported the growth of both *Salmonella* spp. and *E. coli* O157:H7 in coriander during 6 days of storage at 4 °C after combined treatment of 60 ppm SAEW and UVC LED irradiation. Populations of total aerobic bacteria (TAB) and yeast and mold (YM) in lettuce and kale are also increased after combined treatment of 50 ppm ClO_2_ and UVC irradiation after 7 days of storage at 4 °C [39]. However, the growth of *Salmonella* spp. in cherry tomatoes was inhibited by aerosolization with water, 200 ppm chlorine, peroxyacetic acid (80 ~ 400 ppm), or aqueous ClO_2_ (3 ~ 400 ppm) during 3 weeks of storage at 10 °C [17].

TAB decreased for Fuji apples washed with 50 ppm acidic electrolyzed water during 35 days of storage at 10 °C [43]. Massey et al. [44] also reported that electrostatic spray of 2% malic acid and 2% lactic acid could decrease the *E. coli* O157:H7 population in cantaloupe cubes by 1.9 log CFU g^−1^ after 12 days of storage at 4 °C. Combined treatment of aerosolization with 2% malic acid and UVC irradiation could reduce the populations of *E. coli* O157:H7, *Salmonella* Typhimurium, and *Listeria monocytogenes* in fresh-cut lettuces after 15 days of storage at 5 °C [31].

In the present study, EHEC and *S. aureus* populations in strawberries were decreased by the combined treatment during storage at 10 and 15 °C, whereas the growth of both EHEC and *S. aureus* was not inhibited in baby leaves and sliced onions, and the populations of both pathogens were maintained. The behaviors of pathogens could vary depending on the activity of water (a_w_) or the pH of the food, even within the same type of ready-to-eat food [45]. Such differences in EHEC and *S. aureus* growth among tested groups appeared to be due to the different pHs of soft fresh produce tested in this work. Strawberries (3.36 ± 0.03) have lower pH than baby leaves (6.49 ± 0.02) and sliced onion (5.81 ± 0.20). Thus, the proper and effective tool should be applied by considering the characteristics of each food to promote the safety of fresh produce at the market.

### 3.3. Effect of Combined Treatment of Aerosolization and UVC LED Irradiation on Moisture Loss of Soft Fresh Produce during Storage at 10 and 15 °C

**Figure 4:**
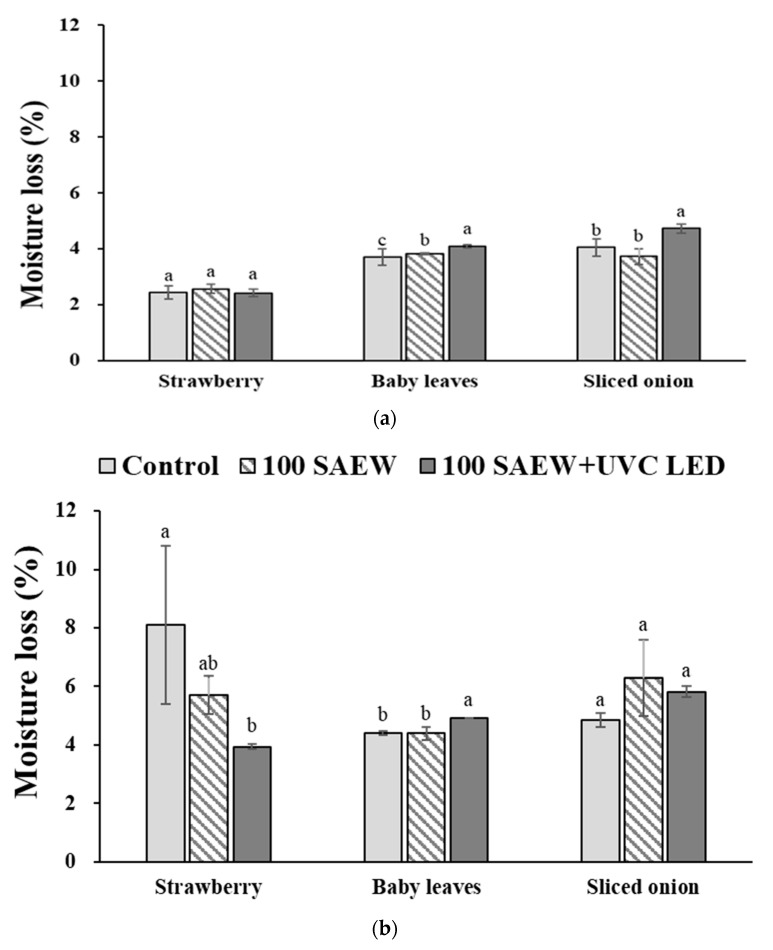
Moisture loss of strawberry, baby leaves, and sliced onion during storage at (**a**) 10 and (**b**) 15 °C for 7 days after applying to inactivate treatment. Control, no extra wash with a sanitizer; 100 SAEW, aerosolization with 100 ppm SAEW; 100 SAEW + UVC LED, aerosolization with 100 ppm SAEW and UVC LED radiation (275 nm). ^a–c^ Different letters represent significant differences measured by Duncan’s multiple tests among treatments within each soft fresh produce at *p* < 0.05.

Overall, significantly lower moisture losses were observed at 10 °C (3.50 ± 0.19%) than at 15 °C (5.38 ± 0.61%) in all samples. The average moisture loss in strawberries, baby leaves, and sliced onion was 2.47%, 3.87%, and 4.16% at 10 °C, respectively, and 5.91%, 4.57%, and 5.65% at 15 °C, respectively (Figure 4). At 10 °C, the moisture loss of strawberries in the 100 SAEW and UVC LED group was not significantly different from that in the control treatment group (Figure 4a). At 15 °C, higher moisture loss was observed for the control group (8.10 ± 2.70%) than for the 100 SAEW and UVC LED (3.94 ± 0.09%), as shown in Figure 4b. On the other hand, significantly higher moisture loss was observed in baby leaves treated with the combined treatment at 10 and 15 °C. In sliced onions, significantly higher moisture loss was only observed with the combined treatment at 15 °C. These results indicate that the combined treatment increased the moisture loss in baby leaves and sliced onions at 10 and 15 °C.

Moisture loss is a major factor related to colour changes of stored strawberries. Moisture loss releases polyphenol oxidase (PPO) enzyme and lowers the concentration of anthocyanin. Thus, moisture loss plays an important role in anthocyanin degradation [46]. Therefore, several studies have investigated precooling, modified atmosphere packaging, and edible coating technologies to prevent moisture loss of strawberries, thus extending their shelf life [47,48,49]. Strawberries with an edible coating (2–3% soy protein-based or 2–3% alginate-based coating) show moisture loss of 4.41% on the 7th day of storage at 4 °C [49]. In the present study, strawberries treated with the combined treatment showed significantly lower moisture losses of 2.42 ± 0.14% and 3.94 ± 0.09% at the 7th day of storage at 10 and 15 °C, respectively. These results indicate that the combined treatment of 100 SAEW and UVC LED could effectively prevent quality changes during cold storage, especially in strawberries.

### 3.4. Effect of Combined Treatment of Aerosolization and UVC LED on Visual Appearance Evolution and Colour Measurement of Soft Fresh Produce during Storage at 10 and 15 °C

**Figure 5:**
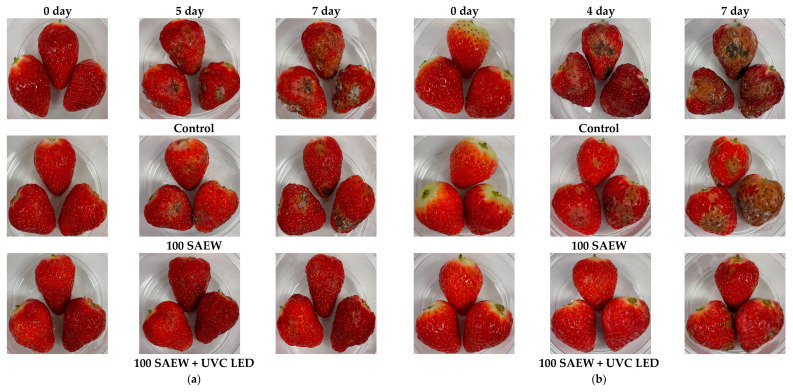
Visual appearance of strawberries stored for 7 days at (**a**) 10 and (**b**) 15 °C. Control, no extra wash with sanitizer; 100 SAEW, aerosolization with 100 ppm SAEW; 100 SAEW + UVC LED, aerosolization with 100 ppm SAEW and UVC LED irradiation (275 nm) for 3 min.

Strawberries treated with the combined treatment showed a better evolution of visual appearance than those that were untreated (controls) or treated with 100 SAEW after 7 days of storage at 10 and 15 °C (Figure 5). At 10 °C, the strawberries from the control group started to show browning on the 4th day of storage, and mold appeared from the 5th day of storage. For strawberries treated with 100 SAEW, mold was also detected from the 5th day of storage. However, strawberries treated with combined treatment showed no mold or browning until the 7th day of storage (Figure 5a). The a* value of strawberries treated with combined treatment on the 4th day was not significantly different from control on day 0 (Table 1).

At 15 °C, mold was observed from the 2nd day of storage. Spoilage of strawberries in the control group progressed from the 4th day of storage. Strawberries from 100 SAEW group showed browning on the surface from the 4th day of storage. In the combined treatment of 100 SAEW and UVC LED group, surface browning of strawberries was noticed from the 6th day of storage. However, mold was not confirmed until the 7th day of storage (Figure 5b). The a* value of strawberries treated with combined treatment on the 4th day was not significantly different from those from the control or treated with SAEW 100 group during 0 day of storage. Furthermore, the a* value of strawberries treated with combined treatment on the 7th day was significantly higher than those from the control or 100 SAEW groups on 4th day (Table 2).

**Figure 6:**
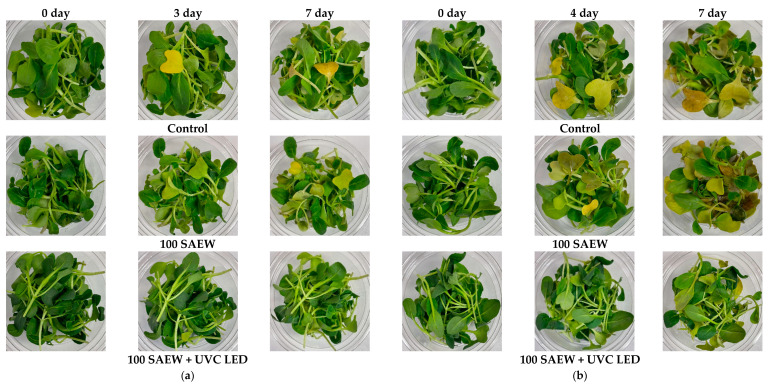
Visual appearance of baby leaves stored for 7 days at (**a**) 10 and (**b**) 15 °C. Control, no extra wash with sanitizer; 100 SAEW, aerosolization with 100 ppm SAEW; 100 SAEW + UVC LED, aerosolization with 100 ppm SAEW and UVC LED irradiation (275 nm) for 3 min.

Baby leaves treated with combined treatment were confirmed to show a better visual appearance than those from the control group or 100 SAEW group during storage at 10 and 15 °C (Figure 6). At 10 °C, leaf discoloration was noticeable for baby leaves in the control group during storage from the 3rd day of storage. In the 100 SAEW group, leaf discoloration began to be observed from the 5th day of storage, whereas the visual appearance of baby leaves in the combined treatment group did not change over 7 days (Figure 6a). At 15 °C, leaf discoloration was noticed for baby leaves after 2–3 days of storage in the control group and the 100 SAEW group. Discolored leaves started to decay and give a foul odor from 4 days of storage. However, baby leaves treated with combined treatment only showed leaf discoloration on the last day of storage (Figure 6b).

**Figure 7:**
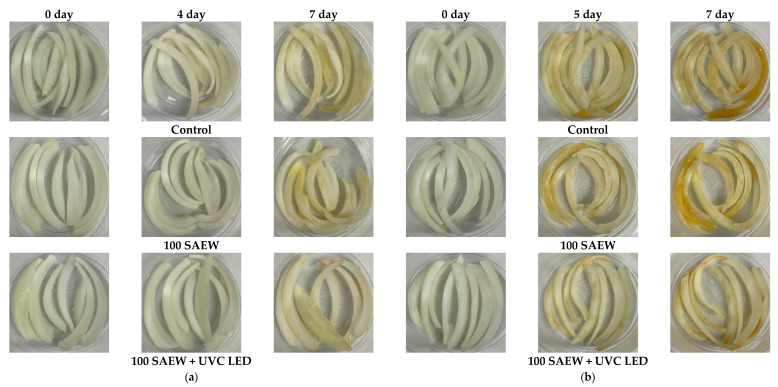
Visual appearance of sliced onions stored for 7 days at (**a**) 10 and (**b**) 15 °C. Control, no extra wash with sanitizer; 100 SAEW, aerosolization with 100 ppm SAEW; 100 SAEW + UVC LED, aerosolization with 100 ppm SAEW and UVC LED irradiation (275 nm) for 3 min.

The L* and b* values of baby leaves in the control group and 100 SAEW group were increased during storage at 10 and 15 °C (Table 1 and Table 2). As yellowish baby leaves occurred in the control and the 100 SAEW groups, these leaves became brighter, indicating an increase in L* and b* values. However, L* and b* values of baby leaves in the combined treatment group were not significantly different from each other at the 4th or 7th day of storage at 10 or 15 °C because leaf discoloration did not occur in this group. However, the b* value of sliced onion treated with combined treatment was significantly different from the control group and 100 SAEW group on the 4th and 7th day of storage at 10 °C (Table 1) and on the 7th day of storage at 15 °C (Table 2). We observed that each treatment affected the color and appearance of sliced onions, and the visual appearance of sliced onions treated with combined treatment was better on the 7th day of storage compared to the other two treatment groups (Figure 7a). At 15 °C, the browning of sliced onions started from day 5 in all treatment conditions, but less damage and decay were observed in the combined treatment group during storage compared to the control group and 100 SAEW group (Figure 7b). The b* value of sliced onions constantly increased during storage at both temperatures in all treatment groups (Table 1 and Table 2). A significant difference in the b* values was noticed among treatments with increased storage time (Table 1).

UVC LED treatment (500 mJ cm^−2^) for apples inoculated with blue mold (*Penicillium expansum*) could prevent the development of blue mold and spoilage during 28 days of storage at 25 °C [50]. UVC treatment could also reduce polyphenol oxidase (PPO) enzyme and improve tissue integrity of fresh-cut Carambola (*Averrhoa carambola* L.), also known as starfruit [40]. Reducing PPO could prevent moisture loss and decrease anthocyanin degradation, avoiding the surface browning in strawberries [46]. The present study also confirmed that strawberries treated with 100 SAEW and UVC LED show the least moisture losses and the greatest visual appearance evolution. The results of this study indicate that UVC treatment has the potential to extend the shelf life of soft fruits such as strawberries. UVC irradiation could also reduce enzyme activity associated with chlorophyll degradation catabolism. Chlorophyll degradation leads a green colour loss in leafy vegetables during chlorophyll degradation; chlorophyll is transformed to chlorophyllide by the chlorophyllase enzyme. Chlorophyllide is then progressed by the enzyme Mg-dechelatase, which removes Mg^2+^ from the chlorophyllide, forming pheophorbide. Consequently, the green colour is lost [41,42,51]. Activities of chlorophyllase and Mg-dechelatase in broccoli and Chinese kale decreased after irradiation with UVC, thus delaying leaf yellowing and showing higher chlorophyll content [52,53]. The results of the present study also show that baby leaves treated with combined treatment of 100 SAEW and UVC LED had less leaf discoloration and showed an improvement of visual appearance. Therefore, combined treatment of SAEW and UVC LED could extend their shelf life in baby leaves at the market by reducing microorganisms and preventing colour change.

## 4. Conclusions

In this study, the efficacy of single (60 SAEW, 100 SAEW, and 100 NaClO) treatment and combined treatment of aerosolization with 100 SAEW and UVC LED (275 nm) for reducing populations of EHEC and *S. aureus* in soft fresh produce was investigated. The highest reductions in EHEC and *S. aureus* populations in strawberries, baby leaves, and sliced onions were observed with the combined treatment of aerosolization of 100 ppm SAEW and UVC LED. The combined treatment further reduced EHEC and *S. aureus* populations in strawberries during 7 days of storage at 10 and 15 °C. Growths of EHEC and *S. aureus* in baby leaves and sliced onions were not inhibited during storage at 15 °C. However, the combined treatment group always showed lower populations than the untreated group (control) during 7 days of storage at 10 and 15 °C. In addition, less moisture loss was observed for strawberries with the combined treatment than for baby leaves and sliced onion during storage at 10 and 15 °C. Overall, the combined treatment showed better maintenance of the color and visual appearance than the untreated group (control) for soft fresh produce during 7 days of storage at 10 °C and 15 °C. Therefore, combined treatment of aerosolization with 100 SAEW and UVC LED could enhance microbial safety and prolong the shelf life of soft fresh produce.

## Figures and Tables

**Table 1:** Effect of combined treatment on L*, a*, b* values in soft fresh produce during 7 days of storage at 10 °C.

Treatments	Storage Time	Strawberry			Baby Leaves			Sliced Onion		
		L*	a*	b*	L*	a*	b*	L*	a*	b*
Control	0 d	33.90 ± 0.86 ^a^	35.17 ± 0.88 ^ab^	15.68 ± 0.54 ^a^	35.26 ± 1.19 ^c^	−9.96 ± 0.46 ^a^	11.18 ± 0.14 ^e^	63.00 ± 1.14 ^a^	−1.07 ± 0.12 ^a^	3.26 ± 0.05 ^d^
	4 d	34.59 ± 1.57 ^a^	24.36 ± 1.23 ^c^	13.11 ± 1.39 ^bc^	43.07 ± 4.01 ^b^	−10.18 ± 0.15 ^a^	15.28 ± 0.80 ^b^	62.85 ± 0.91 ^a^	−1.12 ± 0.13 ^a^	6.25 ± 0.30 ^b^
	7 d	33.84 ± 1.06 ^a^	9.71 ± 0.77 ^d^	11.20 ± 0.20 ^d^	62.74 ± 3.33 ^a^	−9.96 ± 0.54 ^a^	28.87 ± 0.13 ^a^	62.70 ± 0.62 ^a^	−1.14 ± 0.02 ^a^	8.74 ± 0.18 ^a^
100 SAEW	0 d	34.68 ± 0.84 ^a^	35.57 ± 0.47 ^a^	16.10 ± 0.38 ^a^	34.28 ± 2.18 ^c^	−10.07 ± 0.57 ^a^	11.79 ± 0.59 ^de^	62.01 ± 0.40 ^ab^	−1.05 ± 0.10 ^a^	3.36 ± 0.33 ^d^
	4 d	35.06 ± 0.79 ^a^	23.05 ± 4.39 ^c^	13.63 ± 0.79 ^b^	35.60 ± 3.56 ^c^	−9.97 ± 0.34 ^a^	14.79 ± 0.89 ^b^	62.31 ± 0.84 ^a^	−1.14 ± 0.06 ^a^	6.34 ± 0.37 ^b^
	7 d	35.07 ± 0.90 ^a^	9.54 ± 0.90 ^d^	11.76 ± 0.27 ^d^	59.07 ± 1.31 ^a^	−10.52 ± 0.47 ^a^	28.13 ± 1.43 ^a^	61.97 ± 0.54 ^ab^	−1.16 ± 0.16 ^a^	8.66 ± 0.89 ^a^
100 SAEW + UVC LED	0 d	34.57 ± 0.92 ^a^	35.33 ± 0.63 ^a^	15.92 ± 0.72 ^a^	34.10 ± 0.23 ^c^	−10.24 ± 0.36 ^a^	11.08 ± 0.68 ^e^	61.25 ± 1.29 ^ab^	−1.06 ± 0.12 ^a^	3.50 ± 0.20 ^d^
	4 d	34.11 ± 0.39 ^a^	34.17 ± 0.60 ^ab^	13.07 ± 0.37 ^bc^	36.94 ± 0.84 ^c^	−9.96 ± 0.22 ^a^	12.94 ± 0.35 ^cd^	61.72 ± 1.42 ^ab^	−1.09 ± 0.13 ^a^	4.94 ± 0.53 ^c^
	7 d	34.33 ± 0.79 ^a^	31.53 ± 1.10 ^b^	11.76 ± 0.27 ^cd^	35.99 ± 1.74 ^c^	−9.53 ± 0.50 ^a^	13.64 ± 0.89 ^bc^	60.14 ± 0.51 ^b^	−1.24 ± 0.06 ^a^	6.12 ± 0.35 ^b^

L* = lightness, a* = red-greenness, b* = blue-yellowness. Control, no extra wash with sanitizer; 100 SAEW, aerosolization with 100 ppm SAEW; 100 SAEW + UVC LED, aerosolization with 100 ppm SAEW and UVC LED irradiation (275 nm) for 3 min. ^a–e^ Different letters in the same column indicate significant differences measured by Duncan’s multiple tests at (*p* < 0.05).

**Table 2:** Effect of combined treatment on L*, a*, b* values in soft fresh produce during 7 days of storage at 15 °C.

Treatments	Storage Time	Strawberry			Baby Leaves			Sliced Onion		
		L*	a*	b*	L*	a*	b*	L*	a*	b*
Control	0 d	34.57 ± 0.83 ^a^	34.85 ± 0.71 ^a^	16.13 ± 0.63 ^a^	35.62 ± 3.23 ^d^	−9.98 ± 0.38 ^a^	11.67 ± 1.04 ^d^	60.83 ± 1.08 ^a^	−1.04 ± 0.12 ^a^	3.35 ± 0.17 ^c^
	4 d	34.82 ± 0.60 ^a^	16.05 ± 1.82 ^c^	14.07 ± 0.50 ^b^	51.95 ± 0.48 ^b^	−10.06 ± 1.02 ^a^	24.35 ± 1.68 ^b^	60.71 ± 1.14 ^a^	−1.19 ± 0.24 ^a^	7.79 ± 0.29 ^b^
	7 d	35.22 ± 0.19 ^a^	9.40 ± 0.85 ^d^	10.99 ± 0.64 ^c^	63.91 ± 1.26 ^a^	−10.48 ± 0.68 ^a^	31.85 ± 1.33 ^a^	61.42 ± 1.66 ^a^	−1.06 ± 0.08 ^a^	12.07 ± 1.65 ^a^
100 SAEW	0 d	34.43 ± 0.63 ^a^	35.16 ± 0.41 ^a^	16.39 ± 0.86 ^a^	35.19 ± 1.91 ^d^	−9.86 ± 0.29 ^a^	11.24 ± 0.73 ^d^	61.95 ± 1.46 ^a^	−1.06 ± 0.17 ^a^	3.33 ± 0.11 ^c^
	4 d	34.60 ± 0.86 ^a^	18.72 ± 2.05 ^c^	14.08 ± 0.28 ^b^	46.15 ± 0.80 ^c^	−10.43 ± 0.50 ^a^	15.84 ± 0.55 ^c^	61.14 ± 0.85 ^a^	−1.14 ± 0.07 ^a^	7.65 ± 0.48 ^b^
	7 d	34.90 ± 0.42 ^a^	10.33 ± 0.89 ^d^	10.60 ± 0.67 ^c^	61.12 ± 0.39 ^a^	−9.63 ± 0.48 ^a^	26.05 ± 1.81 ^b^	62.48 ± 1.45 ^a^	−1.03 ± 0.09 ^a^	11.41 ± 0.88 ^a^
100 SAEW + UVC LED	0 d	34.71 ± 0.25 ^a^	34.79 ± 0.41 ^a^	16.25 ± 0.25 ^a^	34.01 ± 0.37 ^d^	−9.56 ± 0.21 ^a^	11.26 ± 0.87 ^d^	61.31 ± 0.61 ^a^	−1.13 ± 0.07 ^a^	3.45 ± 0.13 ^c^
	4 d	34.77 ± 0.83 ^a^	34.42 ± 0.62 ^a^	13.94 ± 0.31 ^b^	34.46 ± 1.43 ^d^	−9.96 ± 0.54 ^a^	14.31 ± 0.56 ^c^	62.26 ± 2.71 ^a^	−1.18 ± 0.14 ^a^	6.67 ± 0.18 ^b^
	7 d	34.86 ± 0.11 ^a^	30.59 ± 2.68 ^b^	11.33 ± 0.35 ^c^	35.21 ± 0.63 ^d^	−10.08 ± 0.39 ^a^	15.19 ± 0.43 ^c^	61.56 ± 0.75 ^a^	−1.14 ± 0.08 ^a^	7.27 ± 0.29 ^b^

L* = lightness, a* = red-greenness, b* = blue-yellowness. Control, no extra wash with sanitizer; 100 SAEW, aerosolization with 100 ppm SAEW; 100 SAEW + UVC LED, aerosolization with 100 ppm SAEW and UVC LED irradiation (275 nm) for 3 min. ^a–e^ Different letters in the same column indicate significant differences measured by Duncan’s multiple tests at (*p* < 0.05).

## Data Availability

We did not report any additional data for this study.
